# Casein Glycomacropeptide Hydrolysates Exert Cytoprotective Effect against Cellular Oxidative Stress by Up-Regulating HO-1 Expression in HepG2 Cells

**DOI:** 10.3390/nu9010031

**Published:** 2017-01-15

**Authors:** Tiange Li, Bin Chen, Min Du, Jiajia Song, Xue Cheng, Xu Wang, Xueying Mao

**Affiliations:** 1 Beijing Advanced Innovation Center for Food Nutrition and Human Health, College of Food Science & Nutritional Engineering, China Agricultural University, Beijing 100083, China; 13126750913@163.com (T.L.); songjiajia1208@126.com (J.S.); 2 College of Food Science and Nutritional Engineering, Key Laboratory of Functional Dairy, Ministry of Education, China Agricultural University, Beijing 100083, China; chengxue_604@163.com (X.C.); xuxuwang@foxmail.com (X.W.); 3 Key Laboratory of Space Nutrition and Food Engineering, China Astronaut Training Center, Beijing 100094, China; chenb12@aliyun.com; 4 Department of Animal Sciences, Washington State University, Pullman, WA 99164, USA; min.du@wsu.edu

**Keywords:** casein glycomacropeptide, oxidative stress, HO-1, Nrf2, p38 MAPK, ERK1/2

## Abstract

Oxidative stress is considered as an important mediator in the progression of metabolic disorders. The objective of this study was to investigate the potential hepatoprotective effects and mechanisms of bovine casein glycomacropeptide hydrolysates (GHP) on hydrogen peroxide (H_2_O_2_)-induced oxidative damage in HepG2 cells. Results showed that GHP significantly blocked H_2_O_2_-induced intracellular reactive oxygen species (ROS) generation and cell viability reduction in a dose-dependent manner. Further, GHP concentration-dependently induced heme oxygenase-1 (HO-1) expression and increased nuclear factor-erythroid 2-related factor 2 (Nrf2) nuclear translocation. Moreover, pretreatment of GHP increased the activation of p38 mitogen-activated protein kinase (p38 MAPK) and extracellular signal-regulated protein kinase 1/2 (ERK1/2), which were shown to contribute to Nrf2-mediated HO-1 expression. Taken together, GHP protected HepG2 cells from oxidative stress by activation of Nrf2 and HO-1 via p38 MAPK and ERK1/2 signaling pathways. Our findings indicate that bovine casein glycomacropeptide hydrolysates might be a potential ingredient in the treatment of oxidative stress-related disorders and further studies are needed to investigate the protective effects in vivo.

## 1. Introduction

Oxidative stress may lead to damage to cellular macromolecules such as protein, lipids, and DNA [1]. It has been implicated in the development of several metabolic diseases, including type-2 diabetes, obesity, and non-alcoholic fatty liver disease [2,3]. The excessive generation of reactive oxygen species (ROS) is considered as a main cause of oxidative stress. Thus, how to alleviate the damage of oxidative stress induced by H_2_O_2_ and improve the defensive ability of cells to effectively counteract ROS generation demands more attention. So far, many natural antioxidants from diet supplement, such as dietary polyphenols [4], polysaccharide [5], and peptides [6], have been proposed as preventive or therapeutic agents for oxidative damage caused by ROS. Antioxidant peptides with the characteristics of high antioxidant activity, safety, and absorptivity have generated considerable research interest in food and healthcare fields. Dairy protein is one of the important natural resources to produce antioxidant peptides by enzymatic hydrolysis [7]. These peptides can be served as inhibitors of lipid peroxidation, chelators of transition metal ions, and scavengers of free radicals [8,9]. Casein and whey protein hydrolysates have been shown to possess cytoprotection toward H_2_O_2_-induced oxidative injury via increasing the percentage of viable cells, eliminating intracellular ROS level and activating antioxidant enzymes in vitro [10,11]. However, the exact mechanism underlying the antioxidant activity of protein hydrolysates is not fully understood.

It has been widely accepted that Keap1-Nrf2 pathway is a critical mediator among the mechanisms in defensing against oxidative damages. The redox-sensitive transcription factor nuclear factor erythroid-derived 2 (NF-E2)-like 2 (Nrf2), which can deactivate or eliminate ROS and several carcinogens, is a member of basic leucine-zipper NF-E2 family [12]. Under physiological conditions, Nrf2 is inactive in cytoplasm by association with its inhibitor protein Kelch-like ECH-associated protein 1 (Keap1). To counteract oxidative stress, Nrf2 dissociates from Keap1 before being translocated into the nucleus and then binds to the antioxidant response element (ARE) of cytoprotective genes, which results in the expression of heme oxygenase 1 (HO-1) and other antioxidant enzymes [13]. Nrf2 has been demonstrated to be highly expressed in detoxification organs, especially the liver [14]. Nrf2 knockout mice are extremely sensitive to oxidative damage and lacking in the coordinated gene regulatory program, which indicates the importance of Nrf2 in antioxidant defense [15]. Moreover, accumulating data have verified that many dietary antioxidants possess the ability to selectively activate a series of cellular kinases, including mitogen activated protein kinases (MAPKs). MAPKs which are essential in the regulation of cell proliferation, survival, and apoptosis, have been speculated to involve in the transcriptional activation of Nrf2 [16]. The major types of MAPK subfamilies are p38 MAPK, extracellular signal-regulated kinase (ERK1/2) and c-Jun *N*-terminal protein kinase (JNK1/2). Dietary antioxidants can induce the transcription of phase II antioxidant/detoxifying enzymes through the phosphorylation of MAPKs to defense oxidative injury [17].

Casein glycomacropeptide (GMP) is a 64 amino acid residue C-terminal sialylated phosphorylated glycopeptide derived from κ-casein [18]. GMP and its hydrolysates have deserved much interest for their proposed biological activities, including antibacterial activity, anti-inflammatory activity, promoting bifidobacterial growth, and modulating the immune system response [19,20,21]. In our previous study, GMP hydrolysates (GHP) showed more efficient free radical-scavenging, ferrous ions (Fe^2+^)-chelating and ferric-reducing activity than GMP. Additionally, GHP exerted cytoprotection against oxidative damage in RAW 264.7 macrophages by alleviating ROS generation and enhancing cellular antioxidant enzymes activities via Nrf2/HO-1 pathway [22]. Since liver is the major target organ of toxicants and susceptible to oxidative stress, excessive ROS have also been shown to result in liver injury [23]. The human hepatocyte HepG2 cell line, which retains the endogenous expression of many antioxidant enzymes and other specialized functions, like normal human hepatocytes, has been considered as a good model for investigating cytoprotective mechanisms of natural antioxidants [24]. However, limited studies are available concerning the hepatic cytoprotective effect and its possible mechanism of GHP against oxidative stress.

Therefore, the present study aimed to investigate whether GHP could confer protection against H_2_O_2_-induced oxidative injury in HepG2 cells. To determine the underlying mechanisms of GHP, the defense capacity of GHP on activation of Nrf2 signaling pathway and phosphorylation of MAPKs in HepG2 cells were also investigated.

## 2. Materials and Methods

### 2.1. Materials

GMP with 95% minimum protein content was provided by Arla Co. (Sønderhøj, Viby J, Denmark). Papain (EC 3.4.22.21, from papaya latex, 0.5–2.0 units per milligram), guanidine hydrochloride, methylthiazolyldiphenyl-tetrazolium bromide (MTT), dimethyl sulphoxide (DMSO), and dichloro-dihydro-fluorescein diacetate (DCFH-DA) were obtained from Sigma-Aldrich (St. Louis, MO, USA). H_2_O_2_ was obtained from Beijing Chemical Works (Beijing, China). The bicinchoninic acid (BCA) protein assay kit was obtained from Pierce Chemical Inc. (Rockford, IL, USA). Primary antibodies against Nrf2, ERK, phosphor-ERK, JNK, phosphor-JNK (Thr183/Tyr185), p38 MAPK, phosphor-p38 MAPK (Thr180/Tyr182) were purchased from Cell Signaling Technology (Beverly, MA, USA). Primary antibody against HO-1 was obtained from Abcam (Cambridge, UK). Anti-actin antibody, anti-histone H3 antibody and horseradish peroxidase-conjugated anti-species (mouse and rabbit) secondary antibodies were purchased from Beyotime Institute of Biotechnology (Haimen, Jiangsu, China). All chemical reagents were at least of analytical grade. 

### 2.2. Cell Culture

The human hepatic carcinoma HepG2 cells were purchased from American Type Culture Collection (Rockville, MD, USA) and maintained in minimum essential medium (MEM) supplemented with 10% fetal bovine serum (FBS, Bioin, Israel), 100 U/mL penicillin and 100 μg/mL streptomycin (Invitrogen, Carlsbad, CA, USA). The cells were kept at 37 °C in a humidified 5% CO_2_ atmosphere.

### 2.3. Preparation of GMP Hydrolysate (GHP)

GMP was prepared at a concentration of 5.0% (*w*/*v*) in deionized water. The protein solutions were hydrolyzed using papain with enzyme-to-substrate (E/S) ratio of 5% (*w*/*w*) at optimal conditions (pH 6.0, 55 °C) for 60 min. The pH of hydrolysis was adjusted constant by continuous addition of 1 M NaOH solution to maintain the optimal value. After hydrolysis, the reaction was stopped by heating at 85 °C in a water bath for 20 min to inactivate the protease and then cooled immediately. The hydrolysates were centrifuged at 4000× *g* for 20 min at 4 °C and then the supernatants were collected, lyophilized and stored at −20 °C for further experiments. 

### 2.4. Cell Viability Analysis

Cell viability was measured by 3-(4,5-dimethylthiazol-2-yl)-2,5-diphenyl tetrazolium (MTT) assay. In brief, HepG2 cells were seeded at a concentration of 1 × 10^4^ cells per well in 96-well plates and cultivated with MEM medium for 24 h. Then, cells were incubated with noted concentrations of GHP for 12 h following exposure to H_2_O_2_. Subsequently, 20 μL MTT reagent (5 mg/mL) was mixed with cell cultures for 4 h at 37 °C. The medium was then removed, and the formed formazan was dissolved with DMSO (200 μL). Absorbance was read at 570 nm on a microplate reader (Bio-Rad, Hercules, CA, USA). 

### 2.5. Intracellular Reactive Oxygen Species (ROS) Determination

The generation of intracellular ROS was monitored utilizing DCFH-DA as the fluorescent probe [25]. HepG2 cells were pre-loaded at a concentration of 1 × 10^4^ cells per well in 96-well culture plates. The cells were treated with different concentrations of GHP for 12 h and then stimulated with 400 μM H_2_O_2_ for 30 min. After treatment, cells were washed with PBS to remove GHP and incubated with 50 μM DCFH-DA diluted in MEM for 60 min at 37 °C. Subsequently, the cells were washed three times with PBS and the fluorescent DCF was monitored using a fluorescence-detecting micro-plate reader (Fluoroskan Ascent, Thermo Electron Corporation, Milford, MA, USA) at an excitation wavelength of 488 nm and an emission wavelength of 520 nm. Cells were also collected for each condition and analyzed using a laser confocal scanning system (Zeiss LSM780, Oberkochen, Germany).

### 2.6. Cytosolic and Nuclear Protein Extraction

Cytosolic and nuclear extractions were prepared using a nuclear/cytosol fractionation kit (Biosynthesis Biotechnology Company, Beijing, China). Cells were washed with PBS and harvested with cell lysis buffer. Cell lysates were then centrifuged at 12,000× *g* for 10 min at 4 °C and the precipitates were collected according to the manufacturer’s instructions. Subsequently, the nuclear and cytoplasmic proteins were measured by Western blot. Protein concentration was determined using bicinchonininc acid (BCA) method.

### 2.7. Western Blot Analysis

Cells were washed with PBS and harvested with the treatment of cell lysis buffer (Beyotime Biotech, Haimen, Jiangsu, China) containing 1 mM phenylmethylsulfonyl fluoride (PMSF) (Sigma-Aldrich, St. Louis, MO, USA). Protein concentration was determined using bicinchonininc acid (BCA) method. Equal amounts of protein (20 μg per sample) were subjected to 10% SDS-polyacrylamide gel, followed by electrotransferring to PVDF membranes (Millipore, Billerica, MA, USA). These membranes were then washed with Tris-buffered saline supplemented with 0.05% (*v*/*v*) Tween 20 (TBST) and blocked by 5% (*w*/*v*) skimmed milk powder diluted in TBST. The reactions were incubated with primary antibodies overnight at 4 °C. After incubation, the membranes were washed five times with TBST and then hybridized with secondary antibodies coupled to horseradish peroxidase for 1 h at room temperature. Immunolabeled complexes were detected by enhanced chemiluminescence (ECL) reagents (Millipore, Billerica, MA, USA). Images were obtained by Amersham Imager 600 imaging system (GE Healthcare Life Sciences, Pittsburgh, PA, USA). 

### 2.8. Statistics Analysis

All assays in the present study were performed at least in triplicate and data were expressed as means ± standard deviations (SD). The differences among the groups were analyzed by one-way analysis variance (ANOVA) followed by Duncan’s multiple-comparison test using SPSS software (version 20.0, IBM Inc., Chicago, IL, USA). A *p*-value less than 0.05 was considered statistically significant. 

## 3. Results

### 3.1. Protective Effects of GHP against H_2_O_2_-Induced Cell Injury in HepG2 Cells

#### 3.1.1. Effects of H_2_O_2_ on Cell Viability in HepG2 Cells

**Figure 1:**
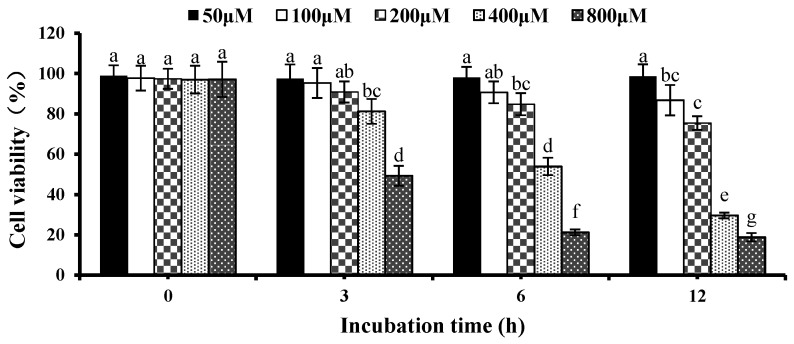
Effects of H_2_O_2_ on cell viability in HepG2 cells. Cells were treated for 3, 6, and 12 h, respectively, with different concentrations of H_2_O_2_. The results are presented as the means ± SD of three independent experiments. Means with different letters are significantly different from each other at *p* < 0.05.

To determine the proper concentration of H_2_O_2_ to induce oxidative stress status, the effect of H_2_O_2_ on cell viability was investigated by MTT assay. HepG2 cells were treated with different concentrations of H_2_O_2_ (0, 50, 100, 200, 400, 800 μM) for 0, 3, 6, and 12 h, respectively. Figure 1 shows that at 6 h, cell viability dropped to 50% with 400 μM H_2_O_2_ compared with the control group without H_2_O_2_, however, 800 μM H_2_O_2_ had a lethal effect on HepG2 cells after co-incubation for 6 h. Therefore, the treatment of H_2_O_2_ 400 μM for 6 h was selected for the following experiments.

#### 3.1.2. Protective Effects of GHP against H_2_O_2_-Induced Intracellular ROS Production in HepG2 Cells

**Figure 2:**
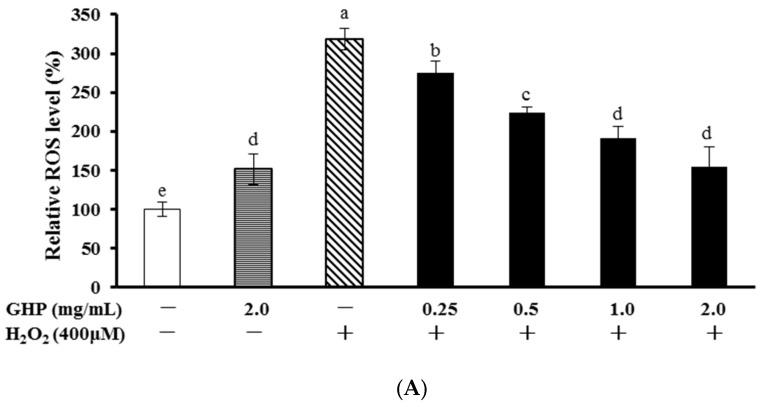

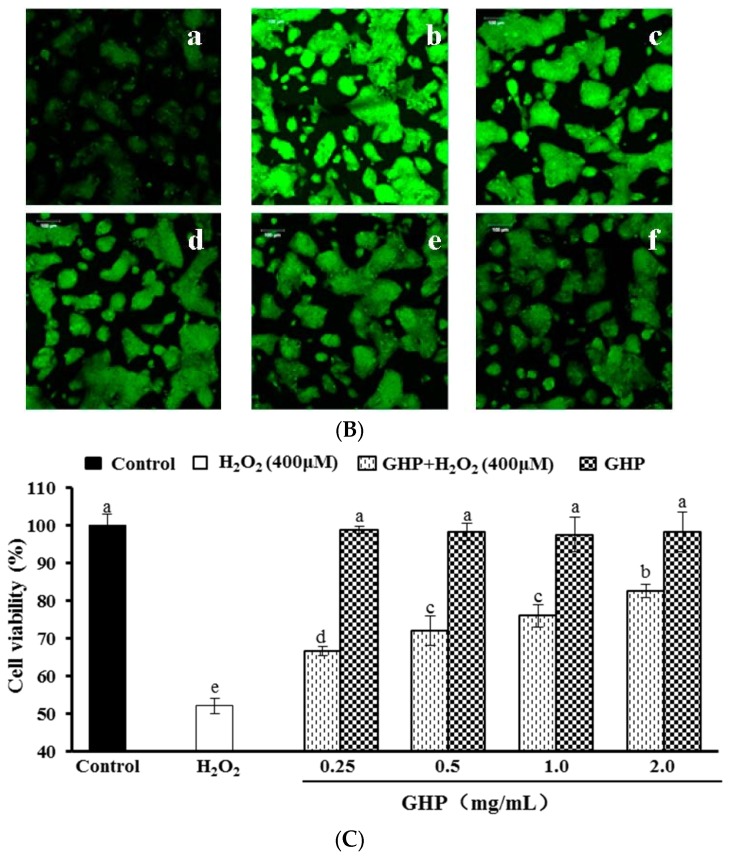
Protective effects of GHP (glycomacropeptide hydrolysates) against H_2_O_2_-induced oxidative stress. (**A**) Cells pretreated with indicated concentrations of GHP for 12 h were stimulated with 400 μM H_2_O_2_ for 30 min. ROS (reactive oxygen species) levels were measured by DCF-DA with fluorescent analysis; (**B**) The ROS levels were analyzed using a confocal scanning system. (**a**) Cells were treated with normal culture medium, (**b**) Cells were treated with 400 μM H_2_O_2_, (**c**–**f**) Cells were pretreated with 0.25, 0.5, 1.0, 2.0 mg/mL GHP, respectively, before 400 μM H_2_O_2_ treatment; (**C**) Cells were treated with the noted concentrations of GHP (0.25–2.0 mg/mL) for 12 h before treatment with 400 μM H_2_O_2_ for 6 h. Cell viability was determined by MTT assay. The results are presented as the means ± SD of four independent experiments. Means with different letters are significantly different from each other at *p* < 0.05.

Intracellular ROS production was measured by monitoring changes in DCF fluorescence to evaluate the antioxidant effect of GHP. As shown in Figure 2A, a mild, but significant, increase in intracellular ROS levels was detected in GHP-treated HepG2 cells. Compared with control cells without H_2_O_2_ addition, H_2_O_2_ treatment increased intracellular ROS accumulation. However, treatment of cells with GHP (0.25, 0.5, 1.0, or 2.0 mg/mL) for 12 h beforehand attenuated H_2_O_2_-induced ROS generation obviously in a concentration-dependent manner. To directly represent the intracellular ROS localization, cells were observed using a laser scanning confocal microscope (Figure 2B). Cells of the control group showed weak green fluorescence, whereas the green fluorescence intensity of H_2_O_2_-treated cells remarkably enhanced, indicating the elevation of intracellular ROS levels. However, this effect was reversed following GHP pretreatment.

The cytotoxicity of GHP on HepG2 cells was determined by MTT assay (Figure 2C). There was no difference on cell viability between GHP-treated and control cells, which indicated that the suppression of GHP on ROS generation was not in relation to a cytotoxic effect in HepG2 cells. In addition, treatment with H_2_O_2_ (400 μM) induced about 50% decline in cell viability, whereas pretreatment with GHP (0.25, 0.5, 1.0, or 2.0 mg/mL) for 12 h remarkably reduced H_2_O_2_-induced decreased cell viability in HepG2 cells. Collectively, these results suggested that GHP possessed protective effects against H_2_O_2_-induced cell injury.

### 3.2. Effects of GHP on HO-1 Expression and Nrf2 Nuclear Translocation in HepG2 Cells

**Figure 3:**
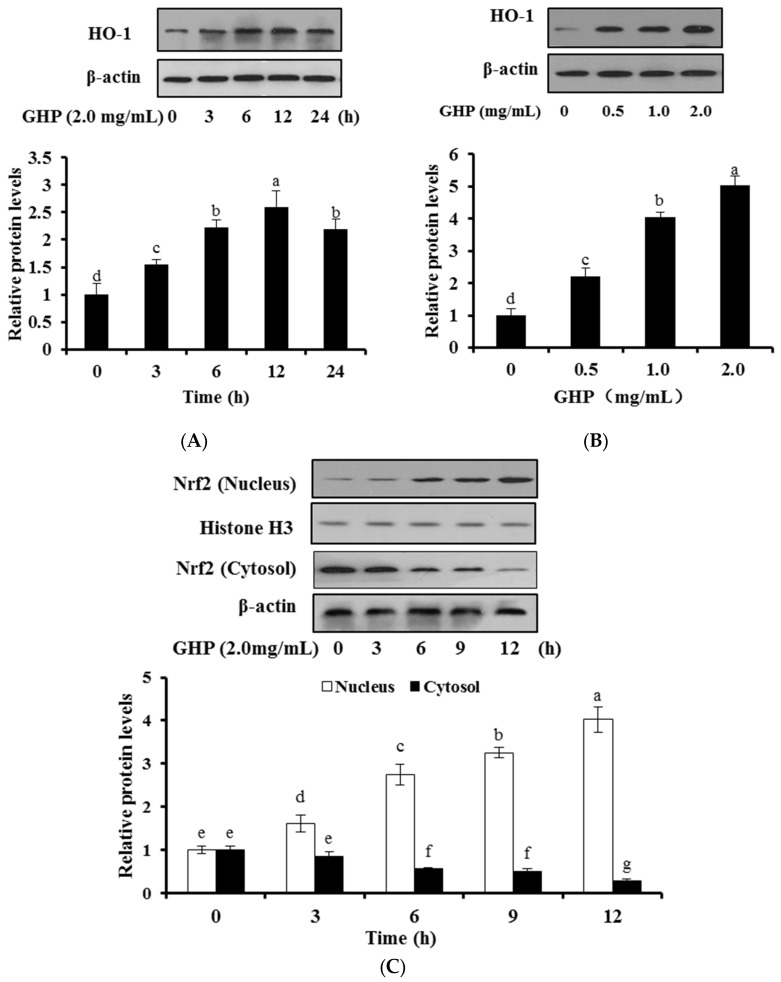
Effects of GHP on HO-1 expression and Nrf2 nuclear translocation in HepG2 cells. (**A**) Cells were treated with GHP (2.0 mg/mL) for indicated time periods; (**B**) Cells were treated with indicated concentrations (0, 0.25, 0.5, 1.0, or 2.0 mg/mL) of GHP for 12 h; (**A**,**B**) Cell lysates were prepared and determined by Western blot to analyze the levels of HO-1; (**C**) Cells were treated with GHP (2.0 mg/mL) for indicated periods. Cytosolic and nuclear fractions were prepared and determined by Western blot to analyze the levels of Nrf2. The results are presented as the means ± SD of three independent experiments. Means with different letters are significantly different from each other at *p* < 0.05.

To explore whether GHP leads to the expression of antioxidant enzymes, the effect of GHP on HO-1 protein expression in HepG2 cells were examined by Western blot analysis. The expression of HO-1 in HepG2 cells was significantly elevated after treatment with GHP (2.0 mg/mL) for indicated periods and reached its summit at 12 h GHP exposure, then, was followed by a decline until 24 h after GHP exposure (Figure 3A). With the maximal effect observed at 12 h, this time point was chosen for further analysis. Treatment of GHP (0, 0.5, 1.0, or 2.0 mg/mL) for 12 h significantly and concentration-dependently up-regulated the expression of HO-1 in HepG2 cells (Figure 3B). 

Then, to investigate whether GHP induces nuclear translocation of Nrf2, the effects of GHP on nuclear Nrf2 protein expression in HepG2 cells at different time intervals (0, 3, 6, 9, or 12 h) were investigated by Western blot analysis (Figure 3C). GHP treatment time-dependently induced the expression of nuclear Nrf2 protein. In contrast, the Nrf2 protein levels in cytoplasm obviously decreased at the first 6 h of GHP exposure. These results indicated GHP promoted the translocation of Nrf2 and the induction of HO-1. 

### 3.3. Activation Effects of GHP on MAPKs in HepG2 Cells

**Figure 4:**
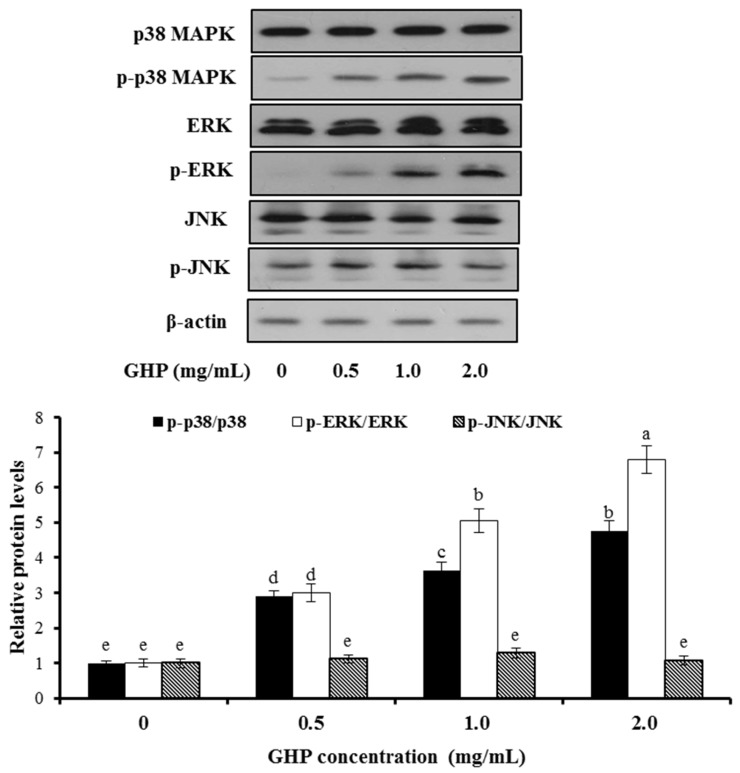
Effects of GHP on phosphorylation and protein expression of ERK1/2, p38 MAPK, and JNK1/2 in HepG2 cells. The activation of the proteins were monitored by Western blot analysis after cells were being treated with GHP (0–2.0 mg/mL) for 1 h. The results are presented as the means ± SD of three independent experiments. Means with different letters are significantly different from each other at *p* < 0.05.

To define the involvement of MAPKs in GHP-mediated Nrf2 activation and induction of HO-1, the phosphorylation and protein expression of MAPK members, including ERK1/2, JNK1/2, and p38 MAPK were assessed by Western blot analysis after HepG2 cells were being incubated with GHP (0, 0.5, 1.0, or 2.0 mg/mL) for 1 h. The present investigation indicated that treatment with GHP markedly up-regulated the protein expression of p-ERK1/2 and p-p38 MAPK in a dose-dependent manner. While the protein expression of p-JNK was not affected and the total protein levels of ERK, JNK, p38 remained unchanged (Figure 4). These findings implied that GHP induced the phosphorylation of ERK1/2 and p38 MAPK, but not JNK1/2.

### 3.4. Roles of p38 MAPK and ERK1/2 Pathways in GHP-Induced Nrf2 Activation and Cytoprotection against H_2_O_2_ in HepG2 Cells

**Figure 5:**
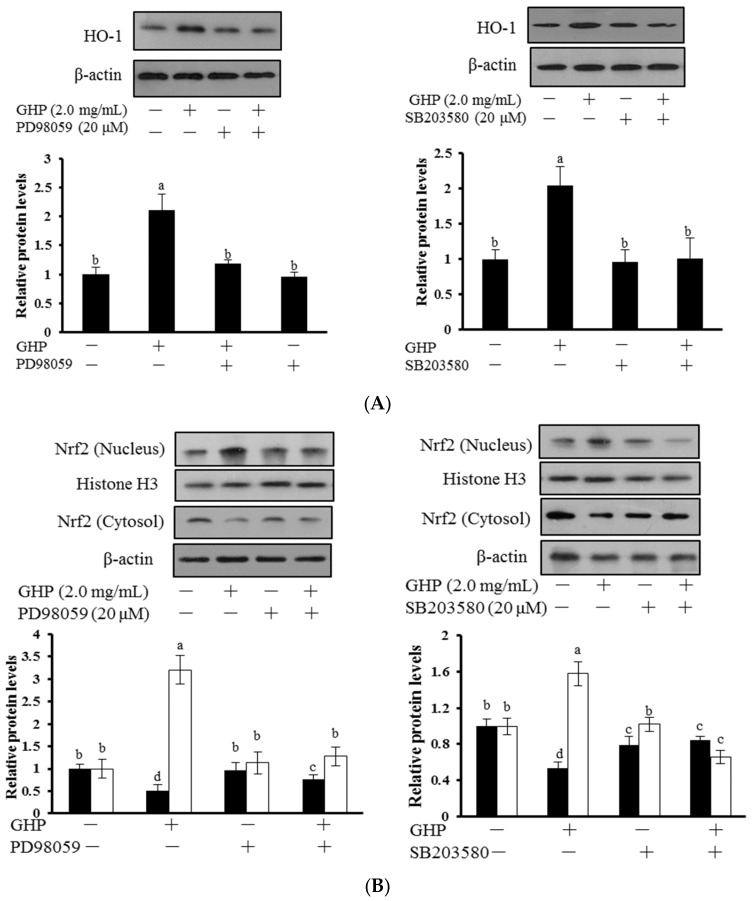
Roles of ERK1/2 and p38 MAPK in GHP-induced Nrf2 activation and HO-1 expression in HepG2 cells. (**A**) Cells were pretreated with PD98059 (20 μM) or SB203580 (20 μM) for 2 h prior to incubation with GHP (2.0 mg/mL) for 12 h. Cells were harvested and HO-1 expression was determined by Western blot analysis; (**B**) Cells were pretreated with either PD98059 or SB203580, each at a concentration of 20 μM, for 2 h prior to incubation with GHP (2.0 mg/mL) for additional 12 h. Then nuclear and cytosol levels of Nrf2 were measured by Western blot analysis. The results are presented as the means ± SD of three independent experiments. Means with different letters are significantly different from each other at *p* < 0.05.

To gain further insight into the molecular mechanisms involved in the cytoprotective effect of GHP, the role of ERK1/2 and p38 MAPK upstream signaling in GHP-induced Nrf2 activation and HO-1 expression were elucidated. Cells were incubated with 2.0 mg/mL GHP for 12 h after pre-incubating for 2 h with SB203580 (an inhibitor of p38) and PD98059 (an inhibitor of ERK1/2), respectively. Then the protein expression level of Nrf2 and HO-1 was determined (Figure 5). GHP treatment induced nuclear Nrf2 accumulation, decreased Nrf2 expression in cytosol, and increased protein expression of HO-1, whereas inhibitors of ERK1/2 and p38 MAPK strongly reduced GHP-induced HO-1 expression (Figure 5A) and nuclear translocation of Nrf2 (Figure 5B). These results indicated that ERK1/2 and p38 were involved in GHP-induced activation of the Nrf2/HO-1 pathway.

**Figure 6:**
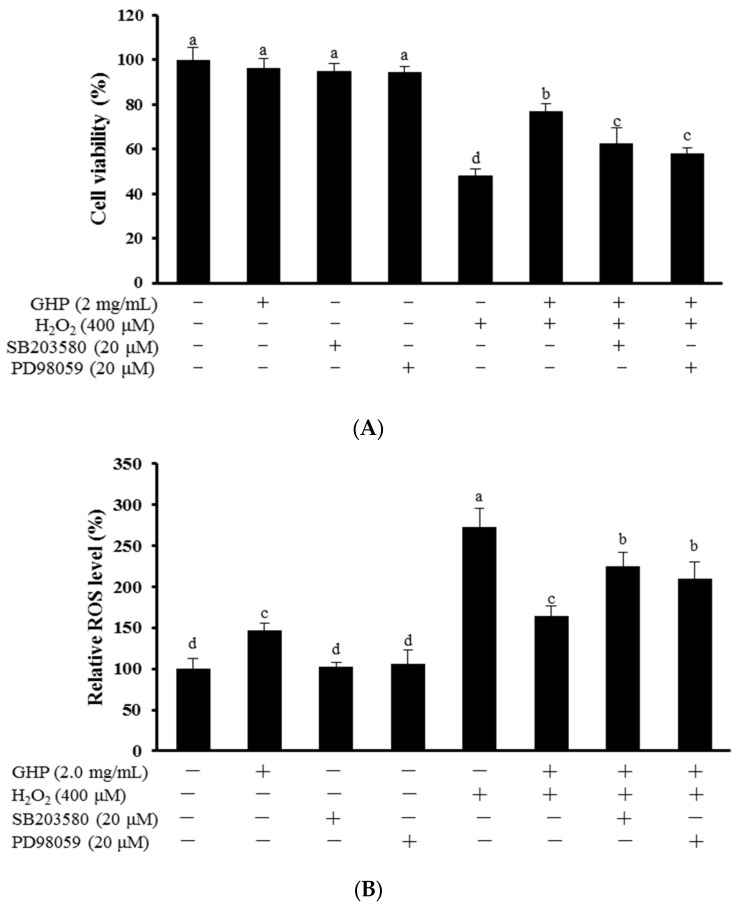
Roles of ERK1/2 and p38 MAPK in GHP-mediated cytoprotection against H_2_O_2_-induced oxidative damage in HepG2 cells. (**A**) Cells were pretreated with PD98059 or SB203580 (20 μM) for 2 h and then incubated with GHP (2.0 mg/mL), or not, for 12 h, followed by stimulation with H_2_O_2_ for 6 h. Cell viability was measured using MTT assay; (**B**) Cells were pretreated with either PD98059 or SB203580 each at a concentration of 20 μM for 2 h prior to incubation with GHP (2.0 mg/mL), or not, for additional 12 h, and then stimulated with H_2_O_2_ for 30 min. The intracellular ROS levels were measured by DCF-DA with fluorescent analysis. The results are presented as the means ± SD of four independent experiments. Means with different letters are significantly different from each other at *p* < 0.05.

Whether the activation of ERK1/2 and p38 contributed to GHP-dependent cytoprotection against H_2_O_2_-induced oxidative stress were further investigated. Cells were pre-incubated with 20 μM SB203580 or PD98059 for 2 h, respectively, and cultured in the presence of 2.0 mg/mL GHP for 12 h. After then, to assess cell viability and intracellular ROS production, GHP-treated cells were exposed to 400 μM H_2_O_2_ for 6 h and 30 min, respectively. As shown in Figure 6A,B, treatment of H_2_O_2_ alone markedly reduced cell viability and induced ROS accumulation, but the effect was obviously reversed after pretreatment of GHP. However, the presence of SB203580 or PD98059 partially abolished the cytoprotection of GHP against H_2_O_2_-induced oxidative stress. These results demonstrated that the protective role of GHP, to some extent, was through the p38 MAPK and ERK1/2 pathway, a crucial upstream signaling pathway that mediated the Nrf2-related cytoprotective effect.

## 4. Discussion

Oxidative stress, which is a condition manifested by excessive generation of ROS, has been linked closely with the progression of several metabolic diseases [2]. Milk protein-derived hydrolysates are considered as important ingredients to fortify the inherent cellular defense capacity against oxidative stress in vitro and in vivo [26]. In this study, we demonstrated that GHP showed the hepatoprotective effects against H_2_O_2_-induced oxidative stress via up-regulation of Nrf2/HO-1 pathway and activation of p38 and ERK1/2 signaling. 

H_2_O_2_, a common form of free radical, has been well characterized to be an oxidative stress inducer because it may evoke intracellular ROS generation in a variety of human cell lines [27]. It passes through cell membranes easily, triggers lipid peroxidation, protein, and DNA damage, which result in cell injury and apoptosis [28]. Many reports have demonstrated that treatment of cells with protein hydrolysates alleviated the H_2_O_2_-induced oxidative damage. Whey protein hydrolysates protected PC12 cells against H_2_O_2_-induced oxidative injury via suppressing ROS elevation and cell apoptosis [29]. An octapeptide from salmon protein showed protection ability against H_2_O_2_-induced DNA damage in Chang liver cells [8]. Pretreatment with GHP was confirmed to significantly increase the cell viability and alleviate the ROS generation in H_2_O_2_-damaged HepG2 cells in our study (Figure 2). Therefore, blocking H_2_O_2_-induced cytotoxicity by GHP may be mediated by an increase in antioxidant capacity, and/or the prevention of oxidative stress. 

The dose of protein hydrolysates reported in the studies on the cytoprotection of protein hydrolysates are usually in the range of 0.5–2.0 mg/mL. The modified casein hydrolysates (0.5–2.0 mg/mL) exhibited cytoprotection on rat hepatocytes in vitro against H_2_O_2_- or galactosamine-induced injury [10]. Wheat germ protein isolate hydrolysates (0.5–1.0 mg/mL) had the protective effects against oxidative stress in PC12 cells [6]. Sodium caseinate hydrolysates at 1.0 mg/mL exhibited genoprotective activity against H_2_O_2_-induced DNA damage in U937 cells [30]. Caseinophosphopeptides (1.0–3.0 mg/mL) protected Caco-2 cells against oxidative damage [31]. Based on these results, we proposed that the concentration of GHP at 0.25–2.0 mg/mL is a reasonable range. However, very few studies have demonstrated the conversion relationship between the doses used in vitro and in vivo. Since the body can absorb, break down and metabolize the hydrolysates in a complex system, in vitro experiments are mainly used to study the mechanism of drug action without being influenced by other factors. Therefore, the dose used in vitro has its limitation and the accurate effective dose in vivo can only be obtained through the animal experiment. 

Numerous studies have demonstrated that antioxidants showed antioxidative activity not only by directly scavenging intracellular ROS, but also by fortifying cellular antioxidant defense system. In this regard, antioxidant and detoxifying enzymes, such as glutathione peroxidase (GSH-Px), catalase (CAT), heme oxygenase (HO-1), NAD(P)H:quinone oxidoreductase I (NQO1) and glutathione *S*-transferase (GST), are important parts of the system [32]. Whey protein hydrolysate mitigated the paracetamol-induced oxidative damage by increasing the total antioxidant potential of hepatocytes and activities of antioxidant enzymes in mice [33]. Casein hydrolysate obtained with Alcalase significantly enhanced CAT activity in oxidative HepG2 cells [34]. In addition, we have previously demonstrated that GHP protected cellular damage by up-regulating the activities of SOD, CAT and GSH-Px in H_2_O_2_-stressed RAW 264.7 macrophages. Among the various cytoprotective enzymes, HO-1 can confer cytoprotection on a wide variety of cells against oxidative damage [35]. Moreover, HO-1 induction has been considered as a crucial beneficial mechanism to maintain homeostasis for the liver injury [36]. In the present study, we found that pretreatment with GHP significantly up-regulated HO-1 expression in a concentration- and time-dependent manner (Figure 3A,B). These results are in agreement with other findings that various natural antioxidants enhanced the endogenous antioxidative defense activity including the induction of HO-1 [37,38]. 

Additionally, the up-regulation of HO-1 is mediated by Nrf2 and the activation of Nrf2/HO-1 pathway can protect cells from oxidative stress-induced damage [39]. Under basal conditions, Nrf2 is anchored to its inhibitory protein Keap1 in the cytoplasm. Upon stimulation, Nrf2 is dissociated from Keap1 following the nuclear translocation and then binds to ARE sites, leading to the activation of ARE-related genes such as HO-1 [40]. In our study, the nuclear translocation of Nrf2 was constantly elevated by GHP (Figure 3C). Similar results were obtained that Nrf2 was involved in the up-regulation of HO-1 to protect hepatic cells against H_2_O_2_-induced cell damage [41].

While high levels of ROS production may result in oxidative stress, increasing evidences have suggested that moderate levels of ROS may function as signals to maintain and promote physiological processes [42]. Bioactive compounds may induce mild oxidative stresses to activate signal transduction pathways, consequently promoting phase II enzyme expression including HO-1 to ameliorate oxidative damage [43]. Epigallocatechin-3-gallate, a dietary polyphenolic compound with high antioxidant activity, upregulated HO-1 expression by increasing ROS production [44]. Consistent with the study, GHP, alone, also caused a slight increase in intracellular ROS production in HepG2 cells.

To further elucidate the underlying molecular mechanism induced by GHP, the activation effects of GHP on mitogen-activated protein kinases (MAPKs) were investigated. The activation of MAPK signaling in HepG2 cells facilitates nuclear translocation and transcriptional activation of Nrf2, thereby induces the antioxidant gene expression, whereas the role of different MAPKs members in Nrf2 activation remains uncertain. The p38 MAPK signaling can be activated in response to cellular stresses, such as oxidative stress or by pro-inflammatory cytokines. ERK1/2 signaling is also commonly involved in cellular protective response to oxidative stress [45]. In this work, we found that GHP addition increased the level of active phosphorylated ERK1/2 and p38 MAPK (Figure 4). One possible explanation for the activation of ERK1/2 and p38 MAPK by GHP may be that GHP, as an inducer of oxidative stress, initiated the intracellular signals and consequently activated Nrf2/HO-1 pathway, resulting in the regulation of redox status against severe oxidative stress. Recently, dieckol, a kind of phlorotannin with high antioxidant activity, activated the Nrf2-ARE signaling pathway through the phosphorylation of ERK1/2, JNK1/2, and p38 MAPK in HepG2 cells [46]. However, geraniin up-regulated the expression of HO-1 by activation of Nrf2-ARE pathways via the phosphorylation of ERK1/2, but not JNK1/2 and p38 MAPK in HepG2 cells [47]. The plausible explanation for the differences on the regulation of Nrf2 signaling pathway may depend on the kinds of inducers. Furthermore, induction of HO-1 and activation of Nrf2 by GHP were inhibited by inhibitors of ERK1/2 (PD98059) and p38 (SB203580), which confirmed that ERK1/2 and p38 MAPK were involved in Nrf2 activation by GHP (Figure 5). Moreover, inhibitors of p38 MAPK and ERK1/2 blunted the cytoprotection of GHP against H_2_O_2_-induced cell death and ROS generation (Figure 6). Taken together, our findings suggest that p38 MAPK and ERK1/2-dependent antioxidant defense pathways might be involved in the cytoprotective effect of GHP. 

Except MAPKs, many other protein kinases can also regulate the activation of Nrf2/HO-1 pathway, such as phosphoinositide 3-kinase (PI3K) [48], AMP-activated protein kinase (AMPK) [49] and protein kinase C (PKCδ) [50]. Thus, it has to be further investigated whether GHP may regulate the activation of Nrf2 via other signaling to play a protective action against oxidative damage. 

## 5. Conclusions

In summary, our study demonstrated that GHP showed hepatoprotective effects against H_2_O_2_-induced oxidative injury in HepG2 cells by increasing cell viability and alleviating ROS generation. GHP induced HO-1 protein expression and facilitated the translocation of Nrf2 into nucleus. Moreover, the upstream signaling pathways, p38 MAPK and ERK1/2, were involved in Nrf2 activation in GHP-stimulated cells and played a critical role in the cytoprotective effect of GHP. These results suggest that GHP may be a potential preventive agent against oxidative injury. As the present study is about the effects of hydrolysates in vitro, further studies are needed to elucidate the protective effect in vivo.
